# Sonic Hedgehog regulates thymic epithelial cell differentiation

**DOI:** 10.1016/j.jaut.2015.12.004

**Published:** 2016-04

**Authors:** José Ignacio Saldaña, Anisha Solanki, Ching-In Lau, Hemant Sahni, Susan Ross, Anna L. Furmanski, Masahiro Ono, Georg Holländer, Tessa Crompton

**Affiliations:** a Immunobiology Section, UCL Institute of Child Health, 30 Guilford Street London WC1N 1EH, UK; b Weatherall Institute of Molecular Medicine, and Department of Paediatrics, University of Oxford, UK

**Keywords:** Thymic epithelium, Morphogen, Sonic hedgehog, T cell, MHCII, mTEC, cTEC

## Abstract

Sonic Hedgehog (Shh) is expressed in the thymus, where it regulates T cell development. Here we investigated the influence of Shh on thymic epithelial cell (TEC) development. Components of the Hedgehog (Hh) signalling pathway were expressed by TEC, and use of a Gli Binding Site-green fluorescence protein (GFP) transgenic reporter mouse demonstrated active Hh-dependent transcription in TEC in the foetal and adult thymus. Analysis of Shh-deficient foetal thymus organ cultures (FTOC) showed that Shh is required for normal TEC differentiation. Shh-deficient foetal thymus contained fewer TEC than wild type (WT), the proportion of medullary TEC was reduced relative to cortical TEC, and cell surface expression of MHC Class II molecules was increased on both cortical and medullary TEC populations. In contrast, the Gli3-deficient thymus, which shows increased Hh-dependent transcription in thymic stroma, had increased numbers of TEC, but decreased cell surface expression of MHC Class II molecules on both cortical and medullary TEC. Neutralisation of endogenous Hh proteins in WT FTOC led to a reduction in TEC numbers, and in the proportion of mature Aire-expressing medullary TEC, but an increase in cell surface expression of MHC Class II molecules on medullary TEC. Likewise, conditional deletion of *Shh* from TEC in the adult thymus resulted in alterations in TEC differentiation and consequent changes in T cell development. TEC numbers, and the proportion of mature Aire-expressing medullary TEC were reduced, and cell surface expression of MHC Class II molecules on medullary TEC was increased. Differentiation of mature CD4 and CD8 single positive thymocytes was increased, demonstrating the regulatory role of Shh production by TEC on T cell development. Treatment of human thymus explants with recombinant Shh or neutralising anti-Shh antibody indicated that the Hedgehog pathway is also involved in regulation of differentiation from DP to mature SP T cells in the human thymus.

## Highlights

• TEC express components of the Hedgehog signalling pathway and transduce it's signals.
• Sonic hedgehog (Shh) is required for normal TEC development.
• Sonic hedgehog particularly influences differentiation to the medullary TEC lineage.
• Shh regulates cell surface MHCII expression on both cortical and medullary TEC.

## 1 Introduction

The thymus provides a specialised environment for the production of T cells. Thymic epithelial cells (TECs) are an essential component of the thymic stroma, and are required to support T cell development. Two broad categories of TEC, which are believed to arise from a common progenitor, have been defined by their localisation, function and cell surface markers [1], [2]. Cortical(c)TEC provide Dl4 for T cell fate specification, and present MHC + peptide ligands for positive selection. They are defined as EpCam1^+^, CD40^+^, CD205^+^, Ly51^+^ and MHCII^+^, and express genes for antigen presentation, including *Cathepsin-L*, *Prss16* and *β5t*. Medullary (m)TEC are specialised for negative selection, and are defined as surface EpCam^+^, CD40^+^, CD205^−^, Ly51^−^ and MHCII^+^ cells that react with the lectin UEA-1. Some mTEC express the *Aire* gene and *Cathepsin-S*, facilitating expression and presentation of Tissue Restricted Antigens for induction of tolerance. While TEC provide multiple essential signals for T cell development, they also require signals from thymocytes for their maturation.

Aire function in mTEC is essential for the induction of tolerance to self in both humans and mice, and Aire mutation leads to profound multi-organ autoimmunity [3], [4]. Other factors which regulate mTEC differentiation and function are also likely to influence self-tolerance, but currently TEC differentiation is not well understood. To date, only a few factors have been identified that are required for TEC differentiation, such as the transcription factor *FoxN1*, which when expressed ectopically can programme other lineages to a TEC fate [5].

During foetal thymus ontogeny, TEC differentiation has been defined in terms of cell surface expression of CD40 and CD205. The TEC progenitor population, which is bipotential for cTEC and mTEC is contained within the CD40^low^CD205^low^ population [6], [7]. Further gradual acquisition of CD40 and increase in CD205 expression gives rise to a transitional progenitor, able to differentiate into either functional cTEC, or into mature mTEC that lose CD205 expression and acquire the expression of mTEC characteristic markers, such as Aire [8], [9]. These observations are consistent with another study which showed that Aire-expressing mTEC originate from β5t^+^ precursors, a molecule expressed in mature cTECs and not in other cell types [10]. Although these markers have proved useful for investigating TEC development, the lineage relationship between mTEC and cTEC populations and the factors that drive the progression from bipotent progenitors, through transitional intermediates, to mature TEC are not well understood in foetal or post-natal thymus [1].

Interactions between TEC and thymocytes have been shown to promote the terminal differentiation of TEC lineages, particularly mTEC, but fate specification to either lineage is believed to occur independently of signals from thymocytes [11]. Relatively few secreted factors or cell–cell interactions have been identified that regulate TEC differentiation, although members of the tumour necrosis factor receptors super family (TNFRSF), including RANK (TNFRST11a), and CD40 and TGF-β are required for normal thymus medulla development, growth and function [12], [13], [14].

Here, we investigate the role of Sonic Hedgehog (Shh) in the regulation of TEC development. Shh is one of three mammalian Hedgehog proteins (Shh, Indian hedgehog (Ihh) and Desert Hedgehog (Dhh)) which share a common signalling pathway. Hedgehog proteins signal by binding to their cell surface receptor Patched1 (Ptch1), and this binding releases Ptch1's repression of Smoothened (Smo), allowing Smo to transduce the Hh signal. At the end of the signalling pathway are the Hh-responsive transcription factors, Gli1, Gli2 and Gli3. *Gli1* is itself an Hh-target gene, and encodes an activator of transcription, whereas Gli2 and Gli3 can be processed to function as transcriptional activators (in the presence of Hh pathway activation) or transcriptional repressors (in the absence of Hh pathway activation). Gli2 is required to intiate the Hh signal, and functions largely as a transcriptional activator *in vivo*, whereas Gli3 functions predominantly as a transcriptional repressor *in vivo*, and can act to repress *Shh* transcription (by repression of an intermediate transcriptional activator) [15]. In fact, in many tissues, Shh and Gli3 have opposing functions, with Shh-deficiency and Gli3-deficiency giving opposing phenotypes [15].

Hedgehog proteins are expressed in the thymus [16], [17], [18], and signal to developing T cells to promote differentiation and proliferation of early thymocyte progenitors [19], [20]. In both mouse and human studies, Hh signalling has been shown to negatively regulate pre-TCR induced differentiation from CD4^−^CD8^−^ double negative (DN) to CD4^+^CD8^+^ (DP) cell [17], [21], [22], [23]. In addition, in mouse studies, Shh has been shown to inhibit TCR-induced differentiation from DP to mature CD4 and CD8 single positive (SP) thymocytes [24], [25], [26], [27]. Sonic hedgehog (Shh) is expressed by thymic stromal cells, and immunofluorescence has located these cells primarily to the cortical medullary junction in mouse and human [17], [18], [20]. Dhh has also been shown to be expressed primarily by stromal cells [18], whereas Ihh is expressed by both stromal cells and thymocytes, with highest expression detected in the DP thymocytes [22].

Given the pivotol role of the thymus in preventing autoimmunity, it is of interest that Hedgehog pathway genes and Hedgehog-pathway targets have been identified in genome wide association studies (GWAS) for the human autoimmune disorder primary biliary cirrhosis, and in primary open-angle glaucoma (POAG), which may be associated with autoimmunity [28], [29]. In addition, components of the Hedgehog-pathway have been detected in sera from patients with Rheumatoid arthritis, Lupus and ankylosing spondylitis [50]. In the human thymus, Hedgehog proteins are expressed by TEC and have been shown to signal to developing thymocyte progenitors, and aberrant Hedgehog pathway activation has been observed in both T acute lymphoblastic leukaemia (T-ALL) and thymoma, which is associated with the neuromuscular autoimmune disorder Myasthenia gravis [18], [23], [30], [31], [32], [33], [34].

Here we analyse the function of Hedgehog signalling in TEC. We show that developing TEC also express components of the Hh signalling pathway and transduce Hh signals in the foetal and adult thymus. We show that Shh is required for normal TEC development, and that it particularly influences the mTEC lineage.

## 2 Materials and methods

### 2.1 Mice

C57BL/6 mice were purchased from Harlan. Gli Binding Site (GBS)-GFP transgenic mice were provided by James Briscoe [35], and *Shh*^+/−^ mice by Phillip Beachy [36], *Shh*F/F and *Gli3*^+/−^ mice were purchased from The Jackson Laboratories.

*Shh*F/F were crossed with *FoxN1-Cre* transgenic mice [37]. Mice were bred and maintained at UCL. All mice studies were reviewed and approved by the British Home Office.

### 2.2 FTOC and culture of human thymus explants

E14.5 Foetal thymi were cultured as described [38] for seven days before analysis. In some experiments, recombinant rHhip (Sigma–Aldrich) was used at 1 μg/ml.

For culture of human thymus explants, human thymus tissue was obtained after surgical removal in children undergoing corrective cardiac surgery. The study had full ethical approval from the Local Research Ethics Committee; in accordance with the Declaration of Helsinki, fully informed consent was obtained from parents of all child donors. The human thymus tissue was cut into ∼1 mm cubed fragments using a scalpel, and cultured on 0.8 μm Millipore filters (Millipore, Massachusetts, US) on 1 ml AIM-V serum free medium (Invitrogen, US) in 24-well plates for five days before analysis. Recombinant Shh protein (at 0.5, 0.25 or 0.125 μg/ml) (R&D Systems) or the anti-Shh neutralising antibody 5E1 (5 μg/ml), as described previously [17], were added where stated.

### 2.3 Isolation of epithelial cells from embryonic and adult thymus

For isolation of adult TEC, thymic lobes were dissected and cut into small pieces (∼10 mm × 10 mm), excess thymocytes removed by sequential washes in HBSS containing FCS 10% (v/v) and incubated for 30 min at 37 °C in HBSS containing FCS, 3.3 mg/ml Liberase and 0.5 mg/ml DNaseI (Roche Diagnostics UK). After incubation, cells suspensions were prepared by gentle mechanical dissociation.

For isolation of embryonic TEC, thymic lobes were dissected and washed once in PBS followed by an incubation of 15 min in PBS containing 0.25% trypsin and 0.02% EDTA and gentle mechanical dissociation to obtain cell suspensions.

### 2.4 PCR analysis for genotyping

DNA for PCR analysis was extracted from tissue as described [39]. Approximately 1 μg of DNA was used as a template in each PCR reaction, using primers as described in Refs. [37] and [19].

### 2.5 Antibodies and Flow cytometry

Cells were stained as described [40], [41], [9] using directly conjugated antibodies against the markers stated (eBioscience; BD PharMingen; and Biolegend, US). Identification of mTEC populations was done using the lectin UEA-1 (Vector Labs, UK). For intracellular Aire staining cells were fixed and permeabilised using the IC fixation/permeabilisation kit (eBioscience).

Experiments were acquired on a Becton Dickinson LSR-II and data representative of >3 experiments analysed using Flowjo 10.6 (Tree Star, US).

### 2.6 Microarray data normalisation

Publicly available gene-expression microarray datasets performed on RNA from wild type highly purified TEC [41] were analysed for the expression of several components of the Hh signalling pathway. Packages “affy” and R were used for quality control and normalisation of the datasets by log-scale Robust Multi-Array analysis (RMA).

### 2.7 Statistical analysis

Statistical analysis was performed using unpaired two-tailed t-tests and probabilities considered significant if P ≤ 0.05 (*), P ≤ 0.001 (**) and P ≤ 0.0001 (***). Data were analysed using Prism 5.0 (GraphPad INC US).

## 3 Results

### 3.1 Hedgehog signalling is active in thymic epithelial cells (TEC) of adult mice

**Fig. 1:**
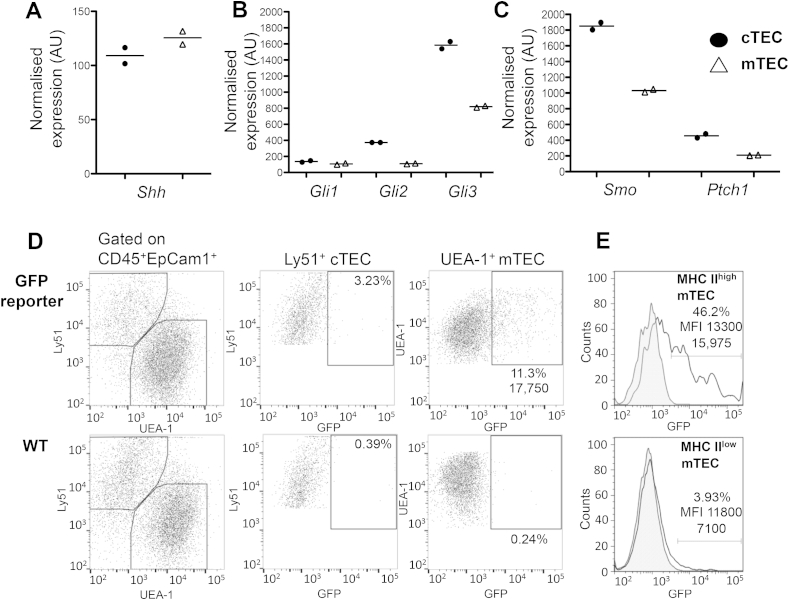
**Hedgehog signalling is active in thymic epithelial cells from adult mice.** (**A–C**) Gene expression by microarray [41] of components of the Hh signalling pathway from sorted cTEC and mTEC extracted from 4 week-old mice. (**D**) Hh signalling in TEC measured by Gli-mediated GFP expression using a reporter transgenic (GBS-GFP-transgenic). (**E**) GFP expression in mature MHCII^high^ and immature MHCII^low^ mTEC. Numbers within plots indicate percentage of GFP positive cells and mean fluorescence intensity (MFI). (**D-E**). Data representative of three independent experiments.

We have previously shown that the components of the Hedgehog signalling pathway are expressed by foetal thymic stroma, [42]. To test if components of the signalling pathway are expressed specifically by TEC, we analysed microarray datasets performed on highly pure sorted TEC populations [41] to assess the expression of *Shh*, the Shh-receptor *Ptch1*, the signal transducer *Smo* and the three Hh-responsive transcription factors (*Gli1*, *Gli2* and *Gli3*). Transcripts for *Shh* and the signal transducers *Gli1-3* were detected both in cortical and medullary TEC (Fig. 1A and B). Transcripts for the transcriptional activator *Gli1* were low in both TEC subpopulations whereas transcripts for *Gli2* and *Gli3*, whose products can act either as transcriptional activators or repressors, were detected at significantly higher concentrations in cTEC when compared to mTEC (Fig. 1B). In addition, *Smo* and *Ptch1* were highly expressed in both TEC subpopulations (Fig. 1C). Taken together, this transcriptional analysis suggested that (autocrine) Shh signalling is likely to occur in TEC.

We next wished to demonstrate active Hedgehog signalling in TEC using a Gli binding site (GBS)-GFP reporter transgenic [35] and determine the proportion of epithelia that display Gli-mediated transcription. Using this transgenic system to measure Hh signaling activity in the adult thymus, we observed that a higher frequency of mTEC showed active signalling (11.3%) when compared to cTEC (3.9%; Fig 1D). This difference is consistent with the higher expression of the transcriptional repressor Gli3 in cTEC (Fig 1B). Most of the mTEC undergoing Hedgehog signalling (GFP+) displayed a mature phenotype, as measured by the expression of high levels of cell surface MHCII, and approximately half of MHCII^high^ mTEC were GFP+ (Fig. 1E). These data thus indicate that adult TEC are undergoing active Hh-signalling, particularly in the mature MHCII^high^ mTEC subset.

### 3.2 Hedgehog signalling is active in developing TEC

**Fig. 2:**
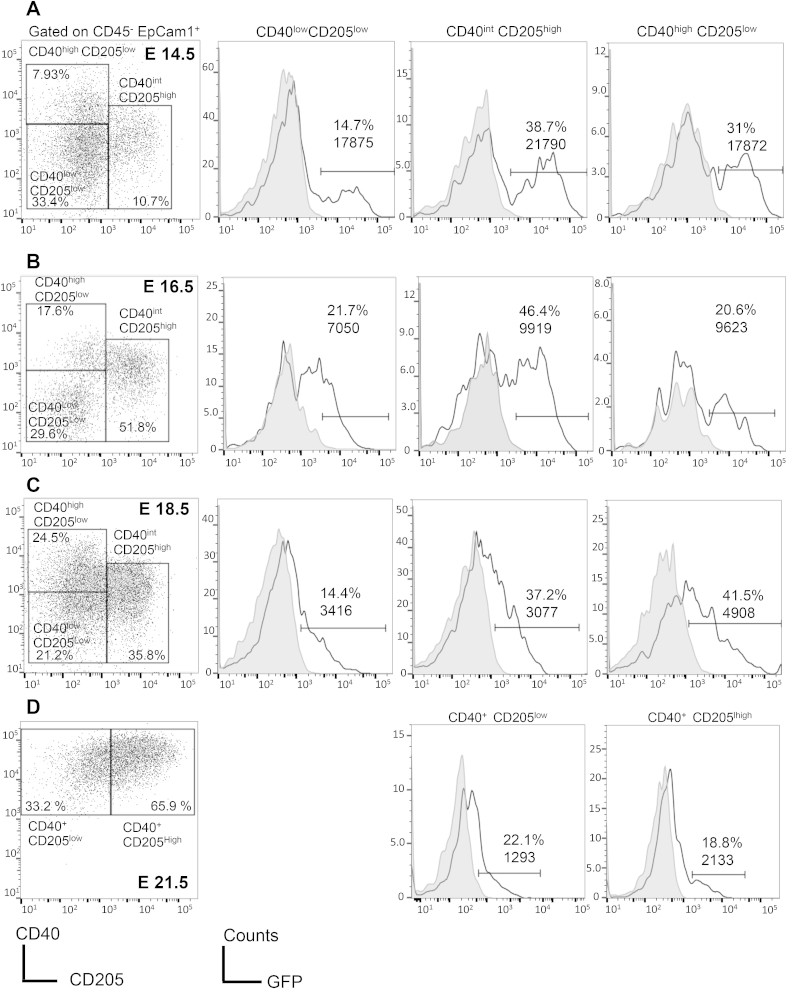
**Hedgehog signalling during foetal TEC ontogeny.** Gli-mediated GFP expression was measured by flow cytometry on the indicated days of embryonic (E) development in GBS-GFP-transgenic thymi. Developing TEC were identified as CD45^−^EpCAM^+^, and stained with anti-CD40 and anti-CD205 to identify three immature TEC populations on E14.5, E16.5 E18.5, and cTEC and mTEC on E21.5 (neonate) (**A–D**): CD40^low^CD205^low^ (containing TEC progenitors), CD40^int^CD205^high^ (immature cTEC) and CD40^high^ CD205^low^ (mTEC lineage); and in neonatal thymus, showing GFP in mTEC and cTEC populations. Open histograms show GFP-fluorescence in a GBS-GFP transgenic thymus and filled histograms WT (background fluorescence). Data representative of three independent experiments.

To assess whether Hh signalling is active in TEC during foetal thymus development, we used the Gli Binding Site (GBS)-GFP reporter transgenic to analyse GFP expression in populations of developing TEC, defined by the changing expression of the developmental markers CD40 and CD205 [8], [9]. We analysed TEC populations on E14.5, E16.5, E18.5 and E21.5 (neonate). We detected GFP expression in all TEC populations, defined by CD40 and CD205 expression (Fig. 2). GFP expression was overall highest in terms of mean fluorescence intensity (MFI) and percentage of positive cells early during development (on E14.5 and E16.5, Fig. 2A and B) with intensity declining through out ontogeny. On E16.5 (Fig. 2B) we found that both CD40^int^CD205^high^ and CD40^high^ CD205^low^ TEC populations expressed similar GFP levels but at the later E18.5 stage, CD40^high^ CD205^low^ TEC expressed higher GFP levels, indicating greater Hh signalling (Fig. 2C). At this time in development, these are cells that are likely to have downregulated CD205 and specified to the mTEC lineage. In the neonatal thymus, the intensity of GFP expression had declined, but GFP was still detectable in both cTEC and mTEC lineages (Fig. 2D). Thus, developing TEC have Gli-mediated transcriptional activity in the foetal thymus, suggesting a role for the Hh pathway during their development.

### 3.3 Foetal TEC development requires Shh

To test whether Shh is required for foetal TEC development, we analysed TEC development in Shh^−/−^ embryos. Shh-deficiency is embryonic lethal, and Shh is expressed by TEC in the foetal and adult thymus (Fig. 1A) [17], [18]. Previous studies have shown that the Shh^−/−^ thymus contains fewer thymocytes than wild type (WT), with inefficient expansion and differentiation of early CD4^−^CD8^−^ (DN) thymocytes [19], [22], but accelerated pre-TCR dependent differentiation from DN to CD4^+^CD8^+^ double positive (DP) thymocyte, and increased efficiency of differentiation from DP to single positive (SP) thymocyte *in vitro* [21], [43]. These defects in thymocyte development could be the consequence of loss of direct Shh-signalling from TEC to thymocytes, or alternatively could result indirectly due to changes in the thymopoietic capacity of TEC, leading to an indirect effect on thymocytes.

**Fig. 3:**
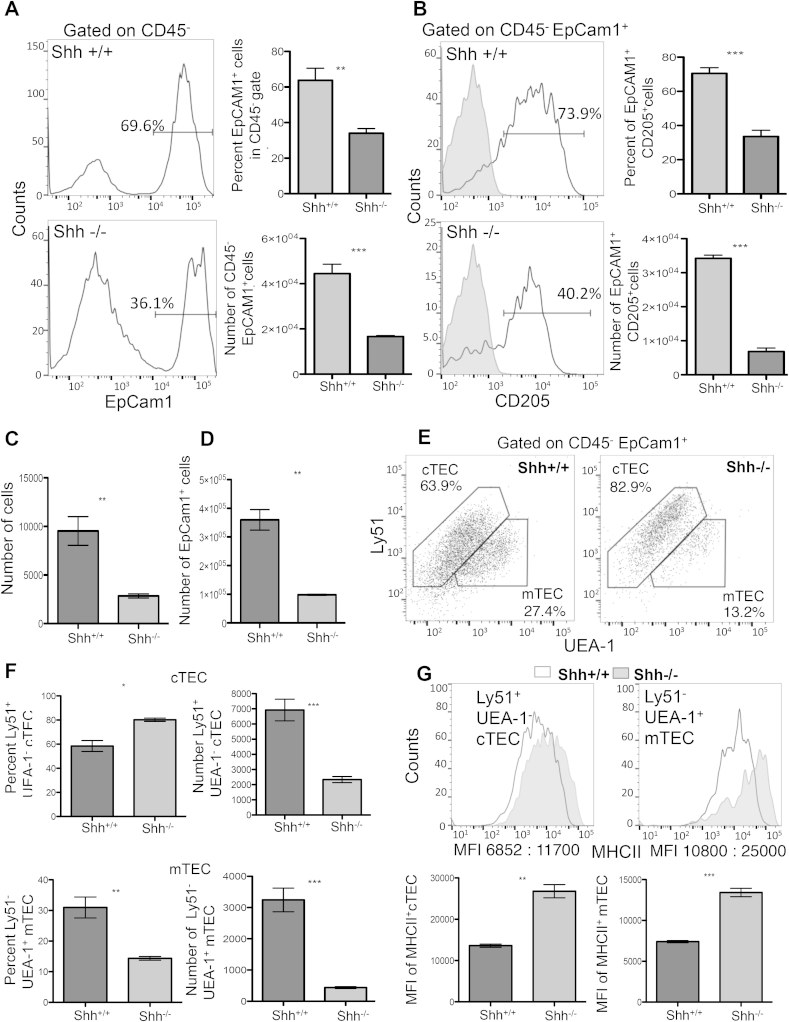
**Reduced TEC differentiation in the E15.5 Shh^−/−^ thymus and Shh^−/−^ FTOC compared to WT**. (**A–B**) TEC populations in E15.5 thymus. (**A**) Histograms show anti-EpCam1 staining on CD45-cells in *Shh*^+/+^ and *Shh*^−/−^ thymus, giving the percentage of cells that stain positive with anti-EpCam1. Bar charts show mean percentage of CD45-cells that stain positive with anti-EpCam1 (upper chart), and number of CD45^−^EpCam1^+^ cells (lower chart). (**B**) Histograms show CD205 expression on CD45^−^EpCam1^+^ cells, and shaded histograms correspond to isotype controls. Bar charts show the mean percentage of EpCam1^+^CD45^−^ cells that stain positive with CD205 (upper chart) and the number of CD45^−^EpCam1^+^CD205^+^ cells (lower chart). N = 4 for both genotypes. Data represent mean ± SD **p < 0.001 ***p < 0.0001. (**C–G**) TEC populations in Shh^−/−^ and WT FTOC. Shh^−/−^ and WT litter mate FTOC were cultured for 7 days and TEC populations were analysed by flow cytometry. (**C**) Number of cells recovered from Shh^−/−^ and WT FTOC. (**D**) Number of epithelial cells (CD45^−^EpCam1^+^) isolated from Shh^−/−^, and WT FTOC. (**E**) Anti-Ly51 (cTEC) and UEA-1 (mTEC) staining on CD45^−^EpCam1+ cells isolated from Shh^−/−^ and WT FTOC, showing the percentage of cells within the region. (**F**) Bar charts show mean percentage of epithelial cells that are cTEC (Ly51+) and mTEC (UEA-1 staining), and the mean number of cTEC and of mTEC isolated from *Shh*^−/−^ and WT FTOC. (**G**) Histograms show cell surface MHCII staining on cTEC and mTEC isolated from Shh^−/−^ (shaded histogram) and WT (open histogram) FTOC. Bar charts show mean of MFI for cell surface MHCII staining on cTEC and mTEC isolated from Shh^−/−^ and WT FTOC.

As *Shh* deletion is embryonic lethal, and most embryos die before E16, we first examined TEC development in the E15.5 foetal thymus. As described previously, the E15.5 *Shh*^−/−^ thymus was smaller than WT [19]. The number of CD45^−^EpCam1^+^ (epithelial lineage cells) was reduced (Fig 3A), and cell surface expression of CD205, and the percentage of CD205^+^ cells within the EpCam1^+^ population were also reduced, relative to WT (Fig. 3B). In order to analyse TEC development in the absence of Shh at later stages of their differentiation, we used foetal thymus organ culture (FTOC) to compare development of *Shh*^−/−^ and WT TEC (Fig. 3C–G). Shh^−/−^ and WT littermate thymus lobes were cultured for 7 days (Fig. 3C–G). The number of cells recovered from Shh^−/−^ thymi was decreased (Fig. 3C) and there was an overall decrease in the number of TEC (CD45^−^EpCam1^+^ cells), relative to WT (Fig. 3D). When we stained against markers of mature cTEC and mTEC populations, Ly51 and UEA-1, we found a significant change in the frequencies of cTEC and mTEC in the Shh-deficient thymus, with a marked reduction in mTEC (Fig. 3E–F). The percentage of mTEC was reduced from 27.4% in the WT FTOC to 13.2% in Shh^−/−^ FTOC, with a concomitant increase in the proportion of cTEC (Fig. 3E). Overall, both the number of cTEC and the number of mTEC were significantly reduced in Shh^−/−^ FTOC compared to WT FTOC, although the mTEC population was affected more severely than cTEC (Fig. 3F). Cell-surface expression of MHCII is essential for both the role of cTEC in positive selection, and the role of mTEC in negative selection, and high cell surface MHCII expression is a marker of mature mTEC. Interestingly, cell surface expression of MHCII was higher in Shh^−/−^ FTOC compared to WT, in both cTEC and mTEC populations (Fig. 3G).

**Fig. 4:**
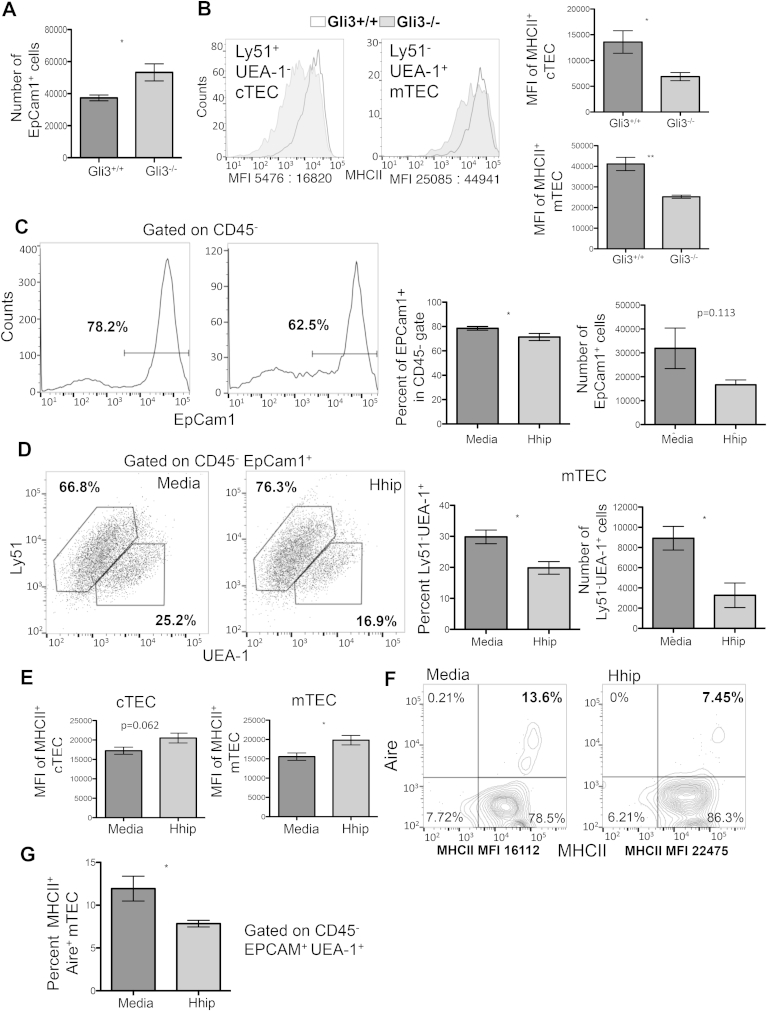
**TEC differentiation in Gli3^−/−^ FTOC compared to WT, and in rHhip-treated WT FTOC compared to untreated. (A**–**B)** Gli3^−/−^ and WT litter mate FTOC were cultured for 7 days and TEC populations were analysed by flow cytometry. (**A**) Bar chart shows mean number of epithelial cells recovered from Gli3^−/−^ and WT littermate FTOC. (**B**) Histograms show cell surface MHCII staining on cTEC and mTEC isolated from Gli3^−/−^ and WT FTOC. Shaded histograms are Gli3^−/−^ and open are WT. Bar charts show mean of MFI for cell surface MHCII staining on cTEC and mTEC isolated from Gli3^−/−^ and WT FTOC. Data represent mean ± SD. N = 5 for Shh^−/−^ and their WT littermate and N = 3 for Gli3 and their WT littermates **p < 0.001 ***p < 0.0001. (**C–G**) **rHhip treatment modulates TEC differentiation in WT FTOC.** TEC development was analysed by flow cytometry in WT FTOC treated with rHhip for 7 days, compared to control untreated FTOC. (**C**) Histograms show anti-EpCam1 staining on CD45^−^ cells, giving the percentage of positive cells. Bar charts show mean percentage and number of EpCam1^+^ cells. (**D**) Dot plots show anti-Ly51 (cTEC) and UEA-1 (mTEC) staining on CD45^−^EpCam1^+^ cells isolated from control and rHhip-treated FTOC. Bar charts show mean percentage and number of Ly51^−^UEA-1^+^ (mTEC). (**E**) Bar charts show mean MFI of cell surface anti-MHCII staining on cTEC (left chart) and mTEC (right chart) from treated and control FTOC. (**F**) Contour plots show cell surface anti-MHCII staining and intracellular anti-Aire staining, gated on CD45^−^EpCam1^+^UEA-1^+^ mTEC, from control and rHhip-treated FTOC, showing the percentage of cells that stained positive for both markers. The MFI of MHCII staining is also given. (**G**) Bar chart shows percentage of mTEC (CD45^−^EpCam1^+^UEA-1^+^) that are positive with intracellular anti-Aire staining and anti MHCII staining. For (**C**), (**D**) and (**E**) data were obtained from n = 7 FTOC and for (**F and G**) from n = 3. Data represent mean ± SD, *p < 0.05.

Gli3 acts as a suppressor of Shh mediated signalling [15] and the Gli3-deficient thymus shows increased Hh-mediated transcription in the thymic stroma [42]. We therefore tested if constitutive loss of Gli3 expression caused changes in TEC cellularity, differentiation and phenotype that are opposite to those observed in Shh-deficient FTOC. We observed an increase in TEC numbers in Gli3^−/−^ FTOC relative to WT littermates (Fig. 4A). In addition, TEC from the Gli3-deficient thymus had lower cell surface MHCII expression in both cTEC and mTEC populations (Fig. 4B).

### 3.4 Neutralisation of Hh signalling influences TEC differentiation in WT FTOC

**Fig. 5:**
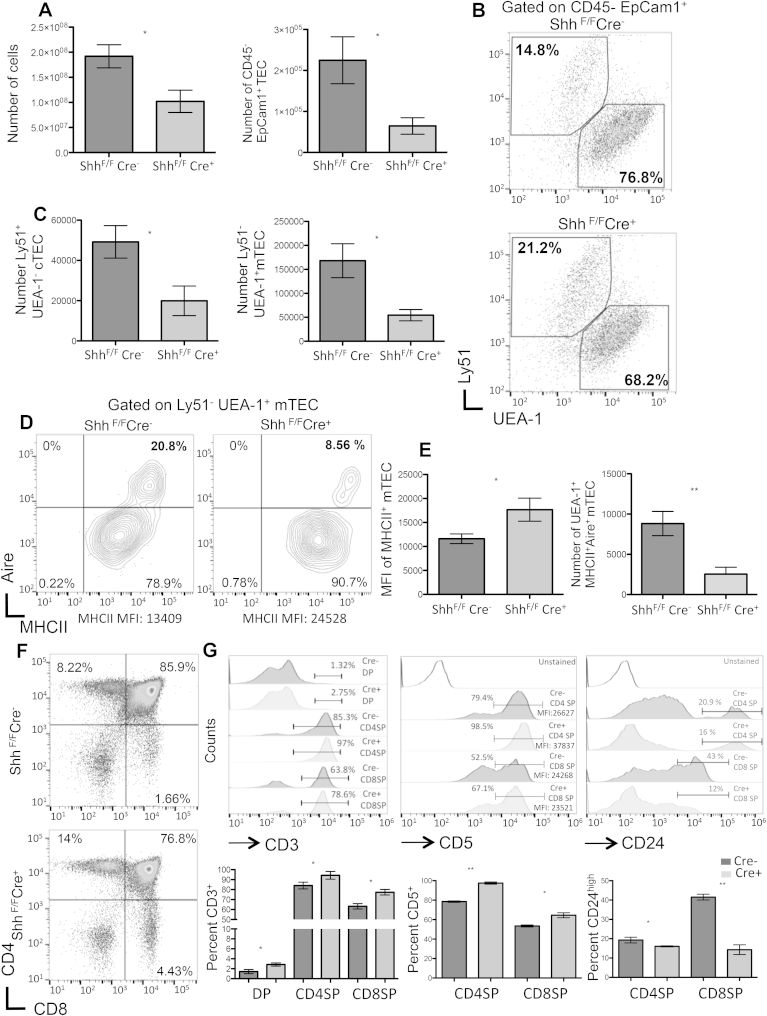
**Conditional deletion of Shh from TEC reduces TEC differentiation in the adult thymus.** Conditional knockout mice of Shh in TEC (Shh^coKO^) were generated by crossing Shhfloxed mice with FoxN1-Cre transgenics to conditionally delete Shh from TEC. Thymus from *Cre*+ (Shh^coKO^) was compared to thymus from *Cre*- (WT) littermates. (**A**) Bar charts show overall number of cells (left) and number of TEC (CD45^−^EpCam1^+^ cells) (right) in the *Cre*- (control) and *Cre+* (*Shh*^coKO^) thymus. (**B**) Dot plots show UEA-1 staining (mTEC) and staining against Ly51 (cTEC), gated on CD45^−^EpCam1^+^ cells, from *Cre*+ and *Cre*-littermates. The percentage of cells within each region is given. (**C**) Bar charts show the mean number of cTEC and mTEC. (**D**) Contour plots show cell surface anti-MHCII staining and intracellular anti-Aire staining, gated on CD45^−^EpCam1^+^UEA-1^+^ mTEC. The percentage of cells in each quadrant and the MFI for anti-MHCII staining are given. (**E**) The bar chart (left) shows the mean MFI of MHCII staining on mTEC and the bar chart (right) shows the mean number of mTEC (CD45^−^EpCam1^+^Ly51-UEA-1^+^) that are positive with intracellular anti-Aire staining. N = 4 mice per genotype. (**F**) Dot plots show anti-CD4 and anti-CD8 staining on thymus from *Shh*^coKO^ and WT mice. Representative percentages of thymocytes from three independent experiments are given. Mean DP:CD4 SP ratio WT = 12.73 ± 1.255, *Shh*^coKO^ = 7.833 ± 1.167 (p = 0.0459) and mean DP:CD8 SP ratio WT = 42.30 ± 4.723, *Shh*^coKO^ = 25.77 ± 3.453 (p = 0.0475). (**G**). Offset histograms represent CD3 (top left), CD5 (top centre), MFI of CD5^high^ SP cells are indicated, and CD24^high^ (top right) expression on thymocytes from *Shh*^coKO^ and WT mice. Bar charts show mean percentage of cells that stain positive with anti-CD3, anti-CD5 and anti-CD24 in the thymocyte subsets. Data represent mean ± SD, *p < 0.05 **p < 0.001 ***p < 0.0001.

Given that Shh is required for normal TEC development in FTOC, we tested if we could manipulate TEC differentiation in WT FTOC by neutralisation of Hh proteins by treatment with soluble recombinant (r)Hhip. Hhip binds to and sequesters Hh proteins [44], and therefore reduces the endogenous Hh signal in FTOC. Treatment of WT FTOC for 7 days with rHhip reduced the proportion of TEC in the CD45^−^ population from 78.2 to 62.5% (Fig. 4D). Within the EpCam1^+^ population, the mTEC lineage (UEA-1^+^) was reduced from 25.2% in untreated FTOC to 16.9% in rHhip-treated FTOC, with a corresponding decrease in mTEC cell number (Fig. 4D). We examined cell surface MHCII expression and found that intensity of staining was significantly increased on the mTEC population (Fig. 5E and F), consistent with the phenotype of *Shh*^−/−^ FTOC (Fig 3G). The MHCII^high^ mTEC population are mature and this population contains Aire-expressing mTEC. Interestingly, although MHCII expression was increased in the rHhip treated cultures, the proportion of mTEC that expressed intracellular Aire protein, declined (Fig. 4F), from 13.6% in control cultures to 7.45% in treated cultures.

Neutralisation of endogenous Hedgehog proteins in FTOC thus had a similar effect to *Shh*-deficiency, as TEC numbers, and the proportion of mTEC were decreased, whereas cell surface MHCII was increased, highlighting the importance of Hh signals for TEC differentiation.

### 3.5 TEC differentiation is reduced in the Shh-deficient adult thymus

To extend our analysis of the role of *Shh* in TEC differentiation and maintenance, we generated mice with a TEC-specific loss of Shh expression. For this purpose we crossed mice transgenic for the expression of the Cre recombinase under the Foxn1 promoter to animals with a conditional *Shh* allele to conditionally delete Shh from TEC lineage cells (Shh^coKO^). The Shh^coKO^ thymus contained fewer cells overall (Fig. 5A), with a profound impact on TEC. There were fewer TEC than WT (Fig. 5A), and numbers of both cTEC and mTEC lineage cells were significantly reduced, although there was an increase in the proportion of cTEC relative to mTEC in the Shh^coKO^ (Fig. 5B–C). Comparable to the findings with foetal Shh^−/−^ FTOC, cell surface expression of MHCII was increased on mTEC, but the proportion of mTEC expressing Aire protein was reduced (Fig. 5D–E).

### 3.6 Influence of conditional Shh-deficiency on thymocyte populations

Constitutive Shh-deficiency has previously been shown to influence T-cell development at the transition from DP to SP in FTOC, increasing the proportion of mature SP thymocytes [24]. However, it has not been possible to use the constitutive Shh^−/−^ mutants to investigate postnatal T cell development given that a constitutive loss of Shh expression results in embryonic lethality, and the influence of conditional deletion of Shh from TEC on thymocyte development is not known. We therefore examined the influence of conditional deletion of *Shh* from TEC in the adult Shh^coKO^ thymus on SP thymocyte differentiation. The proportions of CD4SP and CD8SP cells were significantly increased in the Shh^coKO^ thymus, compared to WT (*Cre*-) littermates, with concommitant significant decrease in the DP population (Fig 5F). The percentage of CD3high cells in the DP, CD4SP and CD8SP populations were higher in the Shh^coKO^ compared to WT thymus (Fig 5G). CD3 is upregulated on DP cells undergoing positive selection, and its high expression in SP populations indicates maturity of the cells, so the higher proportion or CD3high cells suggests that differentiation from DP to mature SP is heightened in the *Shh*^coKO^. Consistent with this, expression of the maturation marker CD24 (HSA), which is downregulated in more mature SP cells, was lower in the Shh^coKO^ populations, with fewer cells falling in the CD24^high^ marker (Fig. 5G). In addition, we measured cell surface CD5 expression. Levels of cell surface CD5 expression on thymocytes are believed to correspond to TCR signal strength [45], and treatment of WT FTOC with rShh has been shown to decrease cell surface CD5 on thymocytes in FTOC [24]. Interestingly, a greater proportion of both CD4SP and CD8SP cells had high levels of cell surface CD5 in the Shh^coKO^ thymus than in WT (Fig. 5G). Overall, the Shh^coKO^ thymus showed an increase in both mature SP populations. Thus, secretion of Shh by TEC is important both for TEC maturation and T-cell development.

### 3.7 Shh regulates differentiation from DP to SP in the human thymus

**Fig. 6:**
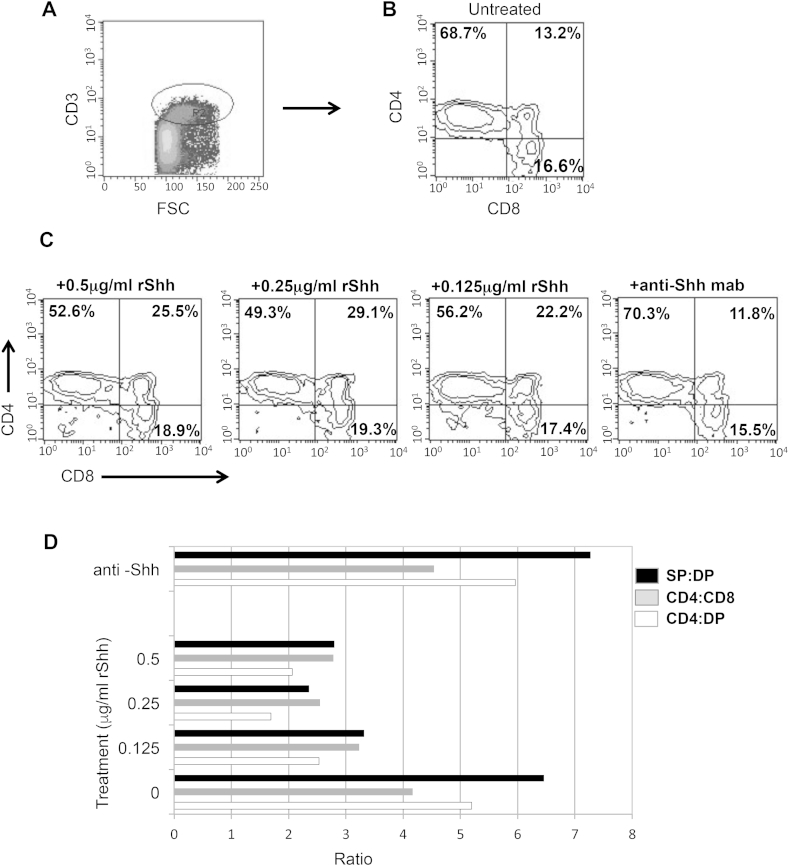
**Shh modulates differentiation from DP to SP thymocyte in the human thymus.** T cell development was analysed by flow cytometry in human thymus explants treated with rShh or neutralizing anti-Shh mab (5E1) for 5 days, compared to control untreated explants. (**A**) Density plot show Forward side scatter (FSC) against anti-CD3 staining, showing the gate on CD3^high^ thymocytes. (**B**) Contour plot shows anti-CD4 and anti-CD8 staining on the CD3^high^ gated population in control untreated thymus explants cultured for 5 days. (**C**) Contour plots show CD4 and CD8 expression in the CD3^high^ gate in thymus explants treated for 5 days with 0.5, 0.25 and 0.125 μg/ml rShh, and 5 μg/ml 5E1 (anti-Shh neutralizing antibody). (**D**) The bar-chart shows the ratios of CD4:DP (open bar), CD4SP:CD8SP (grey bar), and SP:DP (black bar) within the CD3^high^ population, under the different culture conditions. Data are representative of three independent experiments.

Shh is expressed by human TEC [18]. Therefore, in order to test the effect of Shh signalling in human thymocyte differentiation we treated human thymus explants with rShh or neutralising anti-Shh antibody for 5 days, and assessed the SP and DP populations by flow cytometry. Single positive thymocytes accumulated in the thymus explants, and to analyse the mature SP populations, and exclude TCR-negative SP cells that occur before the DP population, we gated on CD3^high^ cells (Fig. 6A) and analysed the percentage of CD4SP, CD8SP and DP within the CD3^high^ gate (Fig. 6B). Treatment with rShh decreased the proportion of CD3^high^CD4SP and CD3^high^CD8SP and increased the proportion of CD3^high^DP cells, with 68.7% of the CD3^high^ gate being CD4SP cells in control-untreated cultures, compared to 49.3% in cultures treated with 0.25 μg/ml rShh. There was a concomitant rise in the DP population, from 13.2% in the control to 29.1% in the rShh-treated cultures (Fig. 6B–C). The proportion of CD4SP cells was more affected by rShh-treatment than proportion of CD8SP cells, so that overall the ratio of CD4:CD8 SP was decreased on rShh treatment, in a dose-dependant manner, as were the ratios of CD4:DP and SP:DP (Fig. 6D). In contrast, when we treated the thymus explants with a neutralising monoclonal antibody against Shh, to neutralise the endogenous Hh proteins in the culture, we found the opposing effect. The accumulation of CD3^high^CD4SP cells was increased compared to untreated cultures, and the ratios of CD4SP:DP, CD4SP:CD8SP and SP:DP were all increased (Fig. 6B–D). Thus, the action of rShh-treatment or neutralisation of endogenous Hh proteins in human thymus explants, mirror the influence of the Hedgehog pathway in the mouse thymus, where increased Hh signalling reduces differentiation from DP to SP cell, and reduction in Hh signalling increases differentiation from DP to SP cell [24], [25], [26], [27], [42].

## 4 Discussion

Here we show that Hedgehog signalling regulates the development of TEC during foetal thymus ontogeny, and also influences TEC production and differentiation in the adult steady-state thymus. The use of GBS-GFP reporter mice, and Shh^−/−^, *Shh*^coKO^ and Gli3^−/−^ mouse mutants confirmed that Shh is required for normal TEC differentiation, that it is produced by TEC and that developing TEC transduce Hh signals.

Interestingly, reduction in Hh signalling to TEC, by *Shh*-deficiency, conditional deletion of Shh from TEC, or neutralisation of endogenous Hh proteins in WT FTOC all led to an increase in cell surface MHCII expression on TEC. Thus, although there are overall fewer TEC in the Shh-deficient thymus, on each individual TEC more selecting MHCII plus peptide complexes for positive and negative selection will be available to developing thymocytes, potentially influencing the outcome of TCR repertoire selection by altering MCHII-restricted TCR antigen dwell time or avidity [46].

Analysis of T cell development in the foetal *Shh*^−/−^ thymus showed increased differentiation from DP to SP cell, whereas treatment of WT FTOC with rShh decreased differentiation from DP to SP, and the CD4:CD8 ratio [24]. In addition, inhibition of *Gli2*-mediated transcription by transgenic expression of a repressor form of *Gli2* in T-lineage cells increased differentiation from DP to SP thymocyte [25], [26], whereas increased Hh-mediated transcription by transgenic expression of an activator form of *Gli2* in T-lineage cells, or by conditional deletion of *Ptch1* from T cells reduced differentiation from DP to SP thymocyte [24], [25], [47]. Activation of Hh-mediated transcription in T lineage cells has been shown to decrease TCR signal strength [24], [48], providing an explanation for the decrease in differentiation from DP to SP on Hh pathway activation and relatively greater impact on the CD4SP lineage on treatment of WT FTOC with rShh. However, our data here suggest that another way in which Shh might impact on differentiation from DP to SP cell is to modulate cell surface expression of MHCII on TEC, influencing signals for positive selection to the CD4 lineage.

Treatment of human thymus explants with rShh or neutralising anti-Shh antibody indicated that Shh also influences differentiation from DP to SP cell, thus underscoring the relevance of these mouse models to human immunity and disease. In both human GWAS studies, patients and experimental animal models, the Hedgehog signalling pathway and its gene targets have been implicated in autoimmunity and allergy [28], [49], [50], [51], [52], [53], but genetic susceptibilities to different autoimmune diseases are complex [54], [55], [56], [57], so in the future it will be interesting to assess the specific contribution of Hedgehog signalling in TEC differentiation and function to the induction and severity of human autoimmune diseases. Interestingly, in primary biliary cirrhosis patients, GWAS have identified both the Hedgehog pathway genes and MHCII genes [54], supporting the idea that the influence of Shh on MHCII expression in the thymus might be important for central tolerance of the CD4 population.

It is important to highlight the influence of Shh on TEC development. Normal differentiation and cellularity of medullary TEC, including the mature MHCIIhigh Aire^+^ mTEC population, is altered in the absence of Shh production by TEC or by neutralization of endogenous Hh signals. These observations are consistent with the role of other morphogens such as TGF-β, which also negatively regulates mTEC maturation and growth, with the adult TGF-β conditional knockout showing a similar proportional change in the mTEC population as we observed in the adult Shh^coKO^ thymus [14]. Gradient signalling by morphogens such as Shh could fine-tune the correct induction of central T-cell tolerance.

## 5 Conclusions

• Few factors that influence TEC differentiation have been identified, and here we show that morphogens, such as Sonic Hedgehog, play an important role in this process.
• Alterations in the Shh signalling pathway have an impact on mTEC development and MHCII surface expression with potential to alter T cell central tolerance.
• It will in the future be important to investigate the impact of Shh signalling to TEC on other known signals required for mTEC development and function such as TNFRST11a and TGF-β, and the way in which Hh pathway activation integrates with RANK or TGF-β signalling.
